# Adaptive Sliding-Mode Controller with Grey Wolf Optimization and Interval Type-2 Fuzzy Logic System for Rehabilitation Lower-Limb Exoskeletons

**DOI:** 10.3390/biomimetics11080546

**Published:** 2026-08-03

**Authors:** Liancheng Zheng, Mohammad Soleimani Amiri, Rizauddin Ramli, Nurul Hamizah Mohamed

**Affiliations:** 1 School of Mechanical Engineering, Shandong Huayu University of Technology, Dezhou 253034, China; p159501@siswa.ukm.edu.my; 2 Department of Mechanical and Manufacturing Engineering, Faculty of Engineering and Built Environment, Universiti Kebangsaan Malaysia, Bangi 43600, Selangor, Malaysia; 3 Faculty of Artificial Intelligence and Cyber Security, Universiti Teknikal Malaysia Melaka, Durian Tunggal 76100, Melaka, Malaysia; nurul.hamizah@utem.edu.my

**Keywords:** interval type-2 fuzzy logic system, lower-limb exoskeleton, grey wolf optimization, sliding-mode controller

## Abstract

In recent years, the potential of exoskeletons to enhance human capabilities has attracted significant research interest. Nevertheless, the control of Rehabilitation Lower-Limb Exoskeletons (RLLEs) is challenging because of their strong nonlinear behaviour. In the paper, a Grey Fuzzy Sliding-Mode (GFSM) controller, which is designed based on the optimization accuracy and estimation capability of the fuzzy logic system, was used for trajectory tracking of a RLLE’s joints. This paper presents the tuning of the controller parameters optimally using Grey Wolf Optimization (GWO) integrated with an Interval Type-2 Fuzzy Logic System (IT2FLS) in real-time. The GFSM was selected as the controller law, in which initially, GWO was used to tune the parameters based on the estimated RLLE’s mathematical model. The optimal tuned parameters were employed to determine the defuzzification range of the fuzzy logic system. IT2FLS was provided to tune the real-time controller parameters. The performance of the GFSM was validated by human-RLLE experiments which showed superior performance compared to other conventional controllers. The experimental results show that the controller achieved reductions in the average error of 81.8%, 82.9%, 84.1%, and 80.6%, respectively, compared with conventional adaptive control methods. These findings indicate that the GFSM can be used to improve motor function recovery in individuals with hemiplegia. By integrating biomechanically inspired motion assistance with IT2FLS, our proposed GFSM controller contributes to the development of biomimetic rehabilitation exoskeletons capable of reproducing natural human gait.

## 1. Introduction

In recent years, spinal cord injuries and strokes have become more prevalent, causing mobility restrictions and balance issues [1,2]. Previous studies show that choosing the right rehabilitation training is essential to reduce the side effects associated with chronic illness [3]. Recent clinical research on neurological rehabilitation indicates that repetitive motor activities positively contribute to the recovery of movement functionality [4]. The repetitive rehabilitation treatment improves the balance of the body equilibrium [5,6]. In classical physiotherapy, treatment generally involves one-to-one, labour-intensive interaction between the therapist and the patient [7]. Conventional hands-on therapy is labour-intensive and presents considerable challenges for physical therapists, particularly when treating a large number of patients [8]. Therefore, the utilisation of rehabilitation devices has increased to improve patients’ motor function and to reduce the physical effort required from physiotherapists by enabling repetitive therapy to prevent muscle atrophy [9,10].

Human locomotion is a highly coordinated biomechanical process involving the synchronized motion of the hip, knee, and ankle joints under the control of the neuromuscular system [11]. Rehabilitation exoskeletons are inherently biomimetic devices, as they are designed to emulate the natural movement patterns of human lower limbs while providing mechanical assistance to restore or enhance mobility. Consequently, the design of the exoskeletons and their control strategy are inspired by the biomechanics of human gait, where coordinated joint motion and adaptive assistance are essential for safe and effective rehabilitation. Therefore, combination of joint actuation and bio-compliant adaptive assistance are essential for safe and effective rehabilitation. By reproducing physiologically compatible trajectories and accommodating variations in human–robot interaction, the control frameworks contribute to the development of biomimetic rehabilitation systems, which can improve rehabilitation performance and enhance the quality of assisted gait via exoskeletons [12].

The application of robots in rehabilitation exercises to support physiotherapists and patients with movement disabilities has been investigated in previous studies [13,14,15]. Patients’ functional impairments are generally in a passive state during the initial stage of rehabilitation [16]. Patients are required to do the exercises with as little activity and energy use as possible. Therefore, exoskeleton robots are employed as rehabilitation devices to support users’ joints and guide limb movement along desired trajectories [17,18]. Lower-limb exoskeletons, as one of the commonly used rehabilitation devices, have been widely utilized to assist patients with disabilities and mobility impairments in performing daily activities [19].

However, attaining precise joint trajectory tracking characterized by robust performance continues to pose a substantial challenge, which is primarily attributable to unknown parameters and uncertainties arising from the individuals and the exoskeleton’s nonlinear characteristics [20,21]. Previous studies have shown that developing robust control techniques capable of handling disturbances while guaranteeing efficient performance across a wide range of medical rehabilitation applications remains challenging [22,23]. Iravani Rad et al. [24] investigated the modelling and experimental validation of a two-degree-of-freedom (DoF) knee exoskeleton for adaptive gait rehabilitation. A Lagrangian-based dynamic model was developed to estimate torque and power requirements, and a fuzzy logic controller was designed. Their method achieved better results on the physical exoskeleton than a traditional control system that used a Proportional–Integral–Derivative (PID) controller and potentiometer sensors. Spyrakos-Papastavridis et al. [25]. presented a novel minimally model-based control framework for flexible-joint robots that enables simultaneous stable trajectory tracking and variable impedance control. Numerical and experimental results validated its effectiveness in achieving stable interaction and tracking performance, allowing flexible-joint robots to replicate biologically inspired behaviours.

In many studies, fuzzy logic control systems have been implemented in exoskeleton control systems due to their strong estimation capability. For instance, Yang et al. [26] proposed a novel adaptive robust control strategy with a fuzzy optimal gain design for a two-DoF lower-limb exoskeleton robot aimed at post-stroke rehabilitation. Their method guarantees system boundedness under uncertainties and disturbances while optimizing control gains using fuzzy set theory, with simulation results confirming improved rehabilitation performance. Lu et al. [27] proposed a low-dimension fuzzy control strategy for a lower-limb exoskeleton robot to address nonlinear dynamics, human–robot interaction complexities, and external disturbances. An explicit dynamic model derived using the Lagrange mean value theorem was combined with a low-dimensional fuzzy estimator for real-time parameter identification, enabling the design of a robust control law. Experimental results on two exoskeleton platforms, tested with both healthy subjects and patients, demonstrated improvements in the proposed control system compared to conventional PID and sliding mode controllers. Li et al. [28] proposed a human operator-involved dynamic locomotion synchronization framework for lower-limb exoskeletons to enhance gait training and movement assistance. A fuzzy-based adaptive controller was developed to track the synchronized trajectory under dynamic uncertainties and input saturation, with experimental results validating its efficacy. Zacharia et al. [29] proposed a sensor-driven neuro-fuzzy control framework based on an Adaptive Neuro-Fuzzy Inference System (ANFIS) for a wearable lower-limb exoskeleton, where motion data from the healthy limb were acquired using flex sensors to generate control signals for the impaired limb. The experimental results demonstrated reliable motion pattern reproduction with low computational cost and practical implementation, highlighting the feasibility of the proposed low-cost exoskeleton as a proof-of-concept rehabilitation system.

Finding optimal controller parameters is challenging, especially for nonlinear dynamic systems [30]. Yusuf et al. [31] developed a model reference adaptive PID controller for a three-DoF lower-limb exoskeleton. They used Grey Wolf Optimization (GWO) to tune the PID controller parameters to initialize their adaptive controller. Li et al. [32] used GWO to identify the dynamic parameters of two-DoF lower-limb exoskeleton that incorporates joint friction. They combined tent chaotic initialization, a nonlinear convergence-factor strategy to improve the performance of GWO for system identification application of the exoskeleton dynamic model. Shadkam et al. [33] compared six widely used meta-heuristic algorithms using an analytical hierarchy process and combined compromise solution method multi-criteria decision-making methods to determine the most effective optimization technique. Their comprehensive evaluation concluded that the Genetic Algorithm (GA) achieved the highest overall ranking, demonstrating superior performance among the considered meta-heuristic algorithms.

Although previous studies such as [24,25,26,27,28,31,32] have successfully employed GWO, fuzzy logic systems, and adaptive sliding mode controllers for exoskeleton control, their integration remains limited. Existing GWO-based methods mainly utilize the optimization algorithm to obtain a single set of controller parameters through offline optimization. Consequently, the optimized parameters remain fixed during operation and cannot adapt to variations in patient dynamics, external disturbances, or modelling uncertainties. On the other hand, fuzzy-based adaptive sliding mode controllers are capable of real-time parameter adjustment; however, their membership functions and parameter ranges are generally designed empirically through expert knowledge or trial and error. Such heuristic designs often limit the controller’s adaptability and reproducibility when applied to different rehabilitation scenarios.

Therefore, a research gap still exists with regard to the development of a systematic framework that connects offline optimization with online adaptive fuzzy control. In particular, there is a need for an approach that employs optimization not only to determine controller gains but also to automatically construct the fuzzy membership function ranges used for real-time adaptation. Addressing this gap can improve controller robustness while eliminating the subjective process of fuzzy membership function design.

Moreover, motivated by the strong estimation capability of fuzzy logic systems, this paper employs a fuzzy logic-based approach to dynamically adjust the parameters of the sliding mode controller in real time. The main contributions of this paper are as follows:

- A hierarchical two-stage Grey Fuzzy Sliding-Mode (GFSM) controller is proposed, in which GWO performs offline optimization while Type-2 Fuzzy Logic System (IT2FLS) performs real-time adaptive tuning of the sliding mode controller.
- Unlike conventional approaches that manually define fuzzy membership functions, the proposed method automatically constructs the IT2FLS membership function boundaries using the optimal parameter ranges obtained from GWO.
- The proposed GFSM enables the controller to preserve online adaptability while constraining the controller parameters within optimization-derived boundaries, thereby improving robustness and tracking performance under nonlinear uncertainties.

To better handle uncertainties and external disturbances, these contributions improved the GFSM control system’s trajectory tracking by dynamically estimating control parameters using the GWO algorithm. Also, the proposed method ensures more effective and reliable performance in Rehabilitation Lower-Limb Exoskeleton (RLLE) applications.

The rest of this article is arranged as follows: In Section 2, an overview of the RLLE is presented and the development of the GFSM control system and system stability are presented. Section 3 represents the validation of the GFSM controller in a RLLE prototype. Section 4 concludes this paper.

## 2. Structure of Optimal Fuzzy Sliding-Mode Controller

### 2.1. Mechanical Structure

**Figure 1:**
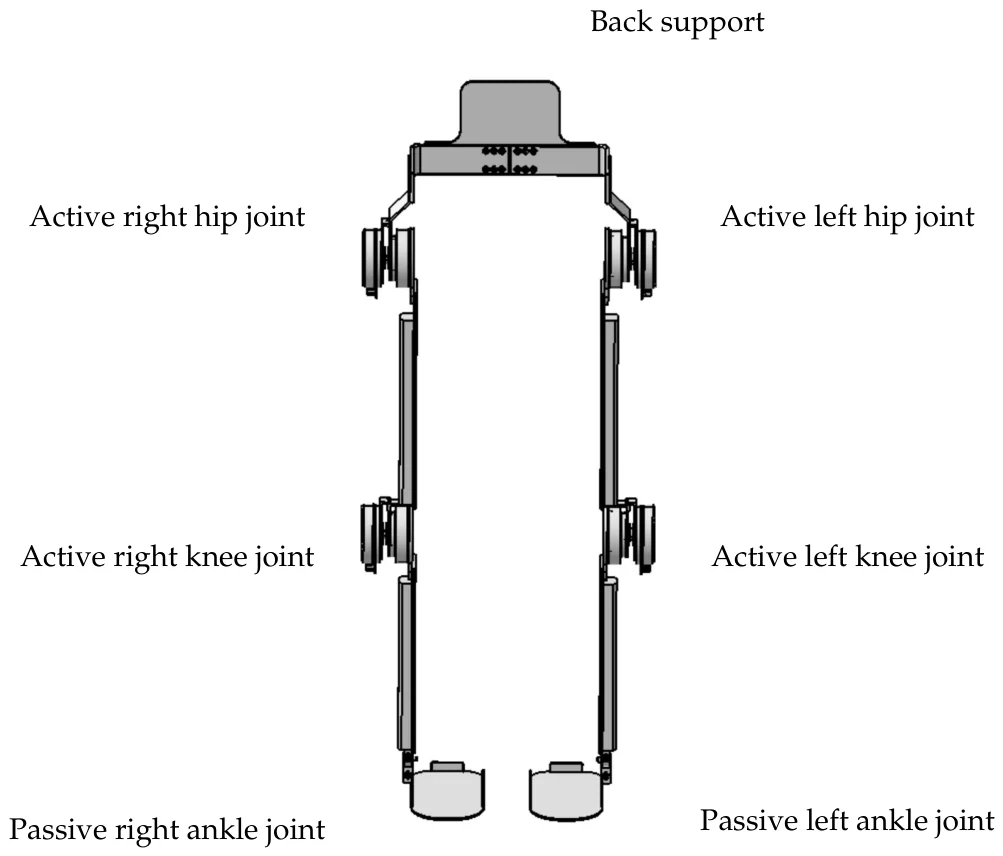
Mechanical configuration and joint arrangement of the proposed RLLE system.

To address the main goal of rehabilitation practice, which is to improve the wearer’s movement ability, a four-DoF RLLE prototype has four active joints on the right/left for hips and knees. The joints are designed for flexion and extension movement of hip and knee joints. Figure 1 shows the mechanical overview of the RLLE and its joint arrangement.

The lengths of the first and second links are 40 cm and 41 cm, respectively. The range of hip joint rotation is from −45 to 30, and the knee joint ranges from −40 to 0 degrees. Table 1 displays the properties of the RLLE.

### 2.2. Mathematical Model

The dynamic model of the RLLE is expressed as follows:

(1) $$M\left(q\right)\ddot{q}+C\left(q,\dot{q}\right)\dot{q}+G\left(q\right)+d\left(t\right)+\tau_{h}=\tau$$

where the joint angle, joint angular velocity, and joint angular acceleration are expressed as *q*, $\dot{q}$, and $\ddot{q}\in {ℜ}^{n}$, respectively. $M\left(q\right)\in {ℜ}^{n\times n}$ is a symmetric and positive definite matrix containing the mass and inertia elements. $C\left(q,\dot{q}\right)\in {ℜ}^{n\times n}$ represents the centripetal and Coriolis forces, $G\left(q\right)\in {ℜ}^{n}$ represents the gravitational force, $d\left(t\right)\in {ℜ}^{n}$ denotes the external disturbance forces, $\tau_{h}\in {ℜ}^{n}$ denotes the interaction torque between the exoskeleton and human, while τ is the force generated by actuators. The inertia matrix M(q), including the distribution of mass and rotational inertia of the links with respect to the joints is given as follows:

(2) $$M\left(q\right)=\left[\begin{array}{cccc} M_{11}\left(q\right) & M_{12}\left(q\right) & \cdots & M_{1n}\left(q\right)\\ M_{21}\left(q\right) & M_{22}\left(q\right) & \cdots & M_{2n}\left(q\right)\\ \vdots & \vdots & \ddots & \vdots \\ M_{n1}\left(q\right) & M_{n2}\left(q\right) & \cdots & M_{nn}\left(q\right) \end{array}\right]$$

The centripetal and Coriolis matrix $C(q,\dot{q})$ is represented as follows:

(3) $$C\left(q,\dot{q}\right)=\left[\begin{array}{cccc} C_{11}\left(q\right) & C_{12}\left(q\right) & \cdots & C_{1n}\left(q\right)\\ C_{21}\left(q\right) & C_{22}\left(q\right) & \cdots & C_{2n}\left(q\right)\\ \vdots & \vdots & \ddots & \vdots \\ C_{n1}\left(q\right) & C_{n2}\left(q\right) & \cdots & C_{nn}\left(q\right) \end{array}\right]$$

where $C_{ij}$ is the elements of $C(q,\dot{q})$ given as follows:

(4) $$C_{ij}=\sum_{k=1}^{n}\frac{1}{2}\left(\frac{\partial M_{ij}}{\partial q_{k}}+\frac{\partial M_{ik}}{\partial q_{j}}-\frac{\partial M_{jk}}{\partial q_{i}}\right){\dot{q}}_{k}$$

The nonlinear state-space representation of the exoskeleton dynamics in Equation (1) is given by

(5) $$\left\{\begin{array}{c} {\dot{X}}_{1}=X_{2},\\ {\dot{X}}_{2}=M^{-1}\left(q\right)\left[\tau -C\left(q,\dot{q}\right)X_{2}-G\left(q\right)-\tau_{h}-d\left(t\right)\right]. \end{array}\right.$$

The nonlinear system in (5) can be compactly written as

(6) $$\dot{X}=f\left(X\right)+g\left(X\right)\tau ,$$

where f(X) and g(X) represent the known nonlinear dynamics, and w(t) denotes lumped uncertainties including human interaction and external disturbances, given as follows:

(7) $$f\left(x\right)=[\begin{array}{c} X_{2}\\ M^{-1}\left(q\right)\left[-C\left(q,\dot{q}\right)X_{2}-G\left(q\right)-\tau_{h}-d\left(t\right)\right] \end{array}]$$

(8) $$[\begin{array}{c} 0\\ M^{-1}\left(q\right) \end{array}]\tau$$

**Assumption 1.**  *The inertia matrix M(q) of the RLLE is symmetric, positive definite, and bounded as follows.*

(9) $$m_{\min}I\leq M\left(q\right)\leq m_{\max}I,\;\forall q$$

*where $m_{\min}$ and $m_{\max}$ are positive constants and $I\in R^{n\times n}$ is the identity matrix. Therefore, the inverse of the inertia matrix M(q) is bounded and satisfies*

(10) $$\frac{1}{m_{\max}}I\leq M^{-1}\left(q\right)\leq \frac{1}{m_{\min}}I$$

**Assumption 2.**  *The desired joint trajectories $q_{d}\left(t\right)$, ${\dot{q}}_{d}\left(t\right)$, and ${\ddot{q}}_{d}\left(t\right)$ are assumed to be bounded and continuously differentiable. That is, there exist positive constants $q_{\max}$, ${\dot{q}}_{\max}$, and ${\ddot{q}}_{\max}$ such that*

(11) $$∥q_{d}\left(t\right)∥\leq q_{\max},\;∥{\dot{q}}_{d}\left(t\right)∥\leq {\dot{q}}_{\max},\;∥{\ddot{q}}_{d}\left(t\right)∥\leq {\ddot{q}}_{\max},$$

*for all t≥0.*

To facilitate the offline optimization of the controller parameters, the nonlinear joint dynamics were represented by equivalent linear transfer-function models identified around the nominal rehabilitation operating conditions.

The dynamic model formulation is adapted from our previous work [34] and is given as follows:

(12) $$G_{1}\left(s\right)=\frac{b_{1}}{a_{3_{1}}s^{3}+a_{2_{1}}s^{2}+a_{1_{1}}s+a_{0_{1}}}$$

(13) $$G_{2}\left(s\right)=\frac{b_{2}}{a_{3_{2}}s^{3}+a_{2_{2}}s^{2}+a_{1_{2}}s+a_{0_{2}}}$$

where $G_{1}\left(s\right)$ and $G_{2}\left(s\right)$ are the transfer functions of the hip and knee joints, respectively. The parameters of the mathematical model for each joint are displayed in Table 2.

Equations (12) and (13), along with the parameters listed in Table 2, were used to define the mathematical model for each joint.

The nonlinear dynamic model in Equation (1) forms the basis for the design of the proposed GFSM controller and the corresponding stability analysis. Since the GWO requires repeated controller evaluations during optimization, directly using the nonlinear model would lead to a high computational cost. Therefore, linear transfer-function models of the hip and knee joints, identified around the nominal rehabilitation operating conditions, are employed only for the offline optimization of the controller parameters. The optimized parameters are subsequently applied to the controller based on the original nonlinear dynamic model and validated through nonlinear simulations and experimental implementation.

### 2.3. Controller Design

In this study, an adaptive sliding mode controller is employed to control the joints of the RLLE. One of the main challenges in controlling nonlinear systems lies in the proper tuning of controller parameters. To address this issue, a fuzzy logic system is utilized to adaptively tune the controller parameters in real time, owing to its strong approximation capability.

A fuzzy logic system is a knowledge-based approach in which the design of membership functions plays a critical role in overall performance. However, defining appropriate membership functions is challenging, as it largely depends on the designer’s experience and expert knowledge.

**Figure 2:**
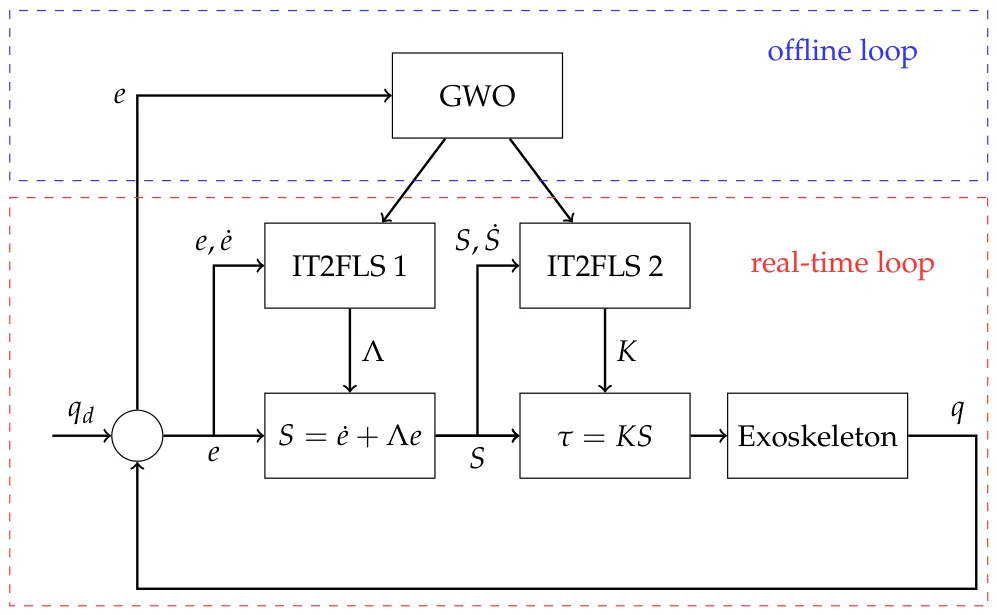
GFSM controller tuning by IT2FLS and GWO.

To overcome this limitation, the tuning of the controller parameters is formulated as an offline optimization problem. The optimal solutions obtained from the optimization process are subsequently used to design the membership functions of the fuzzy logic system. As a result, the proposed control framework combines the advantages of offline optimization and online adaptive control, leading to improved performance and robustness of the LLE system. Figure 2 shows the structure of the GFSM controller tuning by IT2FLS and GWO.

The control law is given as follows:

(14) u=−KS,

where *K* is the sliding gain, which is positive definite, and *s* is the sliding surface defined as

(15) $$S=\dot{e}+\Lambda e,$$

where Λ is the sliding surface slope, which is a positive constant, and *e* denotes the tracking error. The tracking error is defined as

(16) $$e=q-q_{d},$$

where *q* and $q_{d}$ represent the actual and desired joint positions, respectively. The time derivative of the sliding surface is given by

(17) $$\dot{S}=\ddot{e}+\Lambda \dot{e}.$$

The first- and second-order derivatives of the tracking error are given by

(18) $$\begin{array}{cc} \dot{e} & =\dot{q}-{\dot{q}}_{d}, \end{array}$$

(19) $$\begin{array}{cc} \ddot{e} & =\ddot{q}-{\ddot{q}}_{d}. \end{array}$$

From the system dynamics, the joint acceleration is expressed as follows:

(20) $$\ddot{q}=M^{-1}\left(q\right)(\tau -C\left(q,\dot{q}\right)\dot{q}-G\left(q\right)-\tau_{h}-d\left(t\right)),$$

where M(q) is the inertia matrix, $C(q,\dot{q})$ represents Coriolis and centrifugal terms, G(q) denotes the gravitational vector, $\tau_{h}$ is the human interaction torque, and d(t) represents external disturbances. Substituting the system dynamics into the error acceleration yields

(21) $$\ddot{e}=M^{-1}\left(q\right)(u-C\left(q,\dot{q}\right)\dot{q}-G\left(q\right)-\tau_{h}-d\left(t\right))-{\ddot{q}}_{d}.$$

where u=τ is the control input. Hence, the time derivative of the sliding surface can be expressed as

(22) $$\dot{S}=M^{-1}\left(q\right)(u-C\left(q,\dot{q}\right)\dot{q}-G\left(q\right)-\tau_{h}-d\left(t\right))-{\ddot{q}}_{d}+\Lambda \dot{e}.$$

### 2.4. Tuning with Gray Wolf Optimization

In this study, the parameter tuning of the adaptive sliding mode controller is formulated as an optimization problem. To design the objective function, a step response is applied to the transfer function of each joint. The objective function is defined to minimize the tracking error and is formulated as the Root Mean Square (RMS) of the tracking error, given as follows:

(23) $$f_{\mathrm{obj}}=\sqrt{\frac{1}{n}\sum_{i=1}^{n}{∥e_{i}∥}^{2}}$$

where $e_{i}$ denotes the tracking error at the *i*-th sampling instant and *n* represents the total number of samples.

Two controller parameters are considered as design variables, forming a two-dimensional search space for GWO, given by

(24) $$X={\left[\begin{array}{cc} x_{1} & x_{2} \end{array}\right]}^{T}=\left[\begin{array}{cc} K & \Lambda \end{array}\right]$$

where $X\in R^{n}$ is the matrix of the design variables. The optimization problem is designed to determine the optimal values for the controller’s parameters, and it is solved by GWO, which is one of the population-based metaheuristic algorithms inspired by the hunting behaviour of grey wolves [35]. In GWO, candidate solutions represent wolves in a population, and the three best solutions guide the optimization process, which collectively direct the search toward the global optimum, while the remaining wolves update their positions based on these leading solutions [36].

GWO is chosen to tune the controller parameters because it is easy to implement, computationally efficient, and able to search effectively for the best solution [37]. Unlike gradient-based methods, GWO does not need mathematical derivatives, which makes it suitable for nonlinear and complex systems where controller tuning is difficult. GWO gradually converges toward the optimum under the guidance of the three best solutions, which guide the search process. This approach strikes a fair balance between refining promising ones and exploring new solutions, thereby reducing the risk of getting stuck with poor solutions. Additionally, GWO exhibits fast and stable convergence when applied to dynamic systems. Therefore, GWO is a reliable and effective method for optimizing controller parameters and improving closed-loop system performance.

In GWO, candidate solutions represent wolves in a population, and the optimization process is guided by four hierarchical groups, which are the three best solutions, known as α, β, and δ wolves, and ω as the remaining search agents. The three main steps of GWO are encircling, hunting, and attacking the prey.

The hunting behavior of the grey wolf is mathematically modelled by encircling the prey, shown as follows:

(25) $$D=\left|C\times X_{p}\left(i\right)-X\left(i\right)\right|$$

(26) X(i+1)=X(i)−A×D

where $X_{P}$ is the prey position, *X* is the current position vector of the grey wolf and the current iteration is shown by *i*. Coefficient vectors *A* and *C* are given as follows:

(27) $$A=2ar_{1}-a$$

(28) $$C=2r_{2}$$

where $r_{1}$, $r_{2}\in \left[0,1\right]$ are the random vectors. *a* is a linearly decreasing parameter from 2 to 0 over the course of iterations, which controls exploration and exploitation, given by

(29) $$a=2-\frac{2i}{I}$$

with *i* being the current iteration and *I* the maximum number of iterations. Since the exact prey position is unknown, GWO assumes that α, β, and δ wolves have better knowledge of the prey location. The remaining wolves ω follow the three best solutions α, β, and δ. Therefore, the position of each wolf is updated as follows:

(30) $$D_{\alpha }=\left|C_{1}\times X_{\alpha }-X\right|$$

(31) $$D_{\beta }=\left|C_{2}\times X_{\beta }-X\right|$$

(32) $$D_{\delta }=\left|C_{3}\times X_{\delta }-X\right|$$

where $C_{1}$, $C_{2}$ and $C_{3}$ are coefficient vectors and calculated by Equation (28).

(33) $$X_{1}=X_{\alpha }-A_{1}\times D_{\alpha }$$

(34) $$X_{2}=X_{\beta }-A_{2}\times D_{\beta }$$

(35) $$X_{3}=X_{\delta }-A_{3}\times D_{\delta }$$

where $X_{\alpha }$, $X_{\beta }$, and $X_{\delta }$ are the first three best solutions at iteration *i*. $A_{1}$, $A_{2}$, and $A_{3}$ are determined by Equation (27). The final updated position is obtained by averaging:

(36) $$X\left(i+1\right)=\frac{X_{1}+X_{2}+X_{3}}{3}$$

The termination condition of GWO is satisfied when the prey stops moving, and wolves start an attack. Once the termination condition is satisfied, the output of GWO consists of the optimal parameters of the sliding mode controller, *K* and Λ. The algorithm was run for the hip and knee in Equations (12) and (13). The number of wolves was set to five and the maximum number of iterations was set to 500. The GWO algorithm is compared with GA, Particle Swarm Optimization (PSO), and Whale Optimization Algorithm (WOA), which have similar specifications. The population size of GA, PSO and WOA is five and the maximum iteration is 500. Table 3 compares performance in terms of RMS error of the four optimization algorithms, e.g., GWO, GA, PSO and WOA for tuning the controller parameters.

The comparative analysis presented in Table 3 demonstrates that the GWO achieved the best overall tracking performance among the four optimization algorithms. For the hip joint, GWO attained the lowest average RMS tracking error of 0.101 rad, followed by GA, PSO and WOA, PSO, and WOA by 0.114 rad, 0.131 rad and 0.455 rad, respectively. Compared with GA, PSO, and WOA, GWO reduced the average RMS error by approximately 11.4%, 22.9%, and 77.8%, respectively. For the knee joint, GWO also produced the smallest average RMS value of 0.104 rad, outperforming GA, PSO, and WOA followed by 0.113 rad, 0.140 rad and 0.222 rad, respectively. The corresponding improvements were approximately 8.0%, 25.7%, and 53.2%, respectively. Furthermore, the relatively small standard deviations obtained by GWO for both the hip by 0.029 rad and knee by 0.017 rad joints indicate that the optimization results are more consistent and reliable across the ten independent runs compared with the other algorithms, thereby strengthening the robustness of the proposed optimization approach.

In addition to the improved tracking performance, the maximum and minimum values of the optimized parameters *K* and Λ obtained by GWO define a well-distributed range. For the hip joint, *K* varied from 3.09 to 15.10 and Λ varied from 3.53 to 17.83. For the knee joint, *K* ranged from 2.00 to 9.01, and Λ changed from 5.92 to 17.07.

These maximum and minimum ranges of controller parameters are important for defining the interval of the input and output variables in the fuzzy defuzzification stage. This is used to determine appropriate scaling boundaries for the fuzzy system. Compared with GA and PSO, GWO not only produces lower average RMS errors but also provides a more suitable and well-organized range of parameters for fuzzy defuzzification. This contributes to improved controller robustness and overall system stability.

### 2.5. Interval Type-2 Fuzzy Logic System Design

To enhance robustness against uncertainties and nonlinearities, an IT2FLS is employed to adaptively tune the sliding mode controller parameter, *K* and Λ. Unlike conventional Type-1 fuzzy logic, which assigns a precise membership grade to each input, IT2FLS represents uncertainty through upper and lower membership functions. This additional degree of freedom allows the controller to better handle variations in system dynamics and parameter fluctuations that commonly arise in nonlinear control applications [38]. Therefore, the use of interval type-2 fuzzy logic offers a more reliable and flexible parameter-tuning mechanism compared to type-1 fuzzy systems.

In rehabilitation lower-limb exoskeletons, uncertainties arise from multiple sources, including variations in patient body weight, muscle strength, gait characteristics, human–robot interaction forces, and external disturbances. These uncertainties continuously change during rehabilitation sessions and cannot be accurately represented using fixed membership functions. Although Type-1 fuzzy logic systems assign a single membership grade to each input, they are less effective when the uncertainty associated with the input variables changes over time. In contrast, the Footprint of Uncertainty (FOU) of the IT2FLS enables the controller to model uncertainty in the membership functions, resulting in more reliable parameter adaptation and improved robustness under varying rehabilitation conditions.

**Figure 3:**
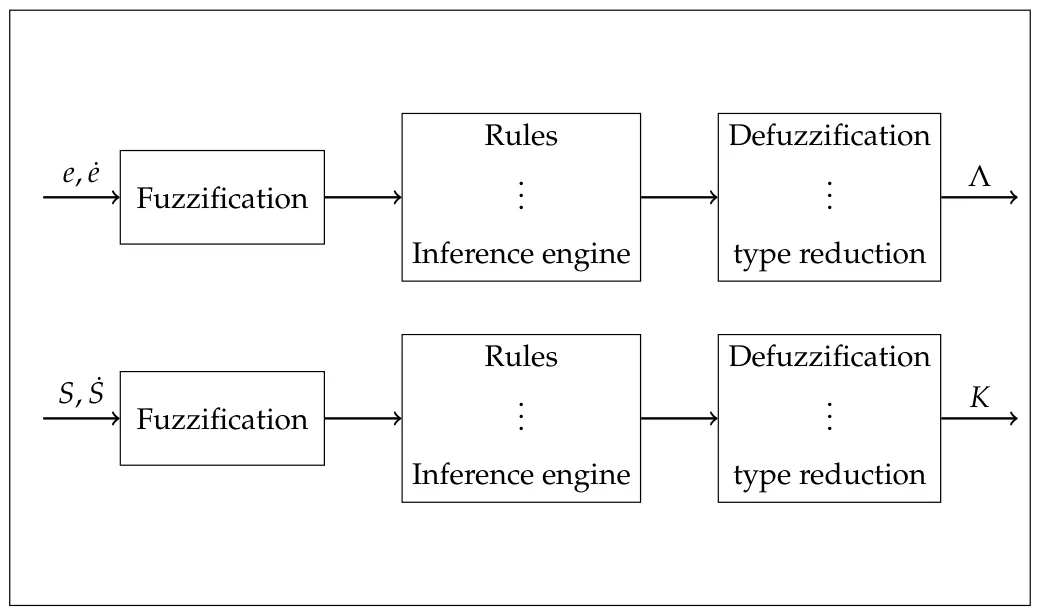
Structure of the IT2FLS.

IT2FLS has two inputs and one output, in which it adjusts the control gain online based on the tracking error and its rate of change, enabling improved performance under varying operating conditions. Figure 3 illustrates the structure of the IT2FLS.

The inputs of the IT2FLS are selected as the tracking error *e* and the derivative of the error $\dot{e}$, while the output of the fuzzy system is the adaptive sliding mode parameter Λ. Also, *S* and $\dot{S}$ are the inputs to the second fuzzy system to adjust parameter *K* of the controller.

The input vector of the fuzzy system is given as follows:

(37) $$x\left(t\right)=\left[e,\dot{e},S,\dot{S}\right]$$

**Figure 4:**
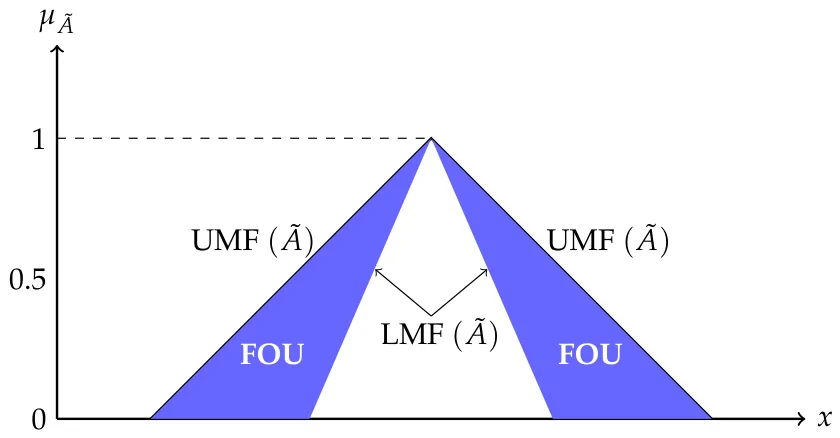
Lower and upper membership function of IT2FLS.

In IT2FLS, the fuzzification stage converts the crisp signal into interval type-2 fuzzy sets. Each membership function is characterized by an upper and a lower triangular function. An interval type-2 fuzzy set $\tilde{A}$ is defined as:

(38) $$\tilde{A}=\left\{\left(x,\;[{\underline{\mu }}_{A}\left(x\right),{\bar{\mu }}_{A}\left(x\right)]\right)|x\in X\right\}$$

where ${\underline{\mu }}_{A}\left(x\right)$ is the lower membership function (LMF), ${\bar{\mu }}_{A}\left(x\right)$ is the upper membership function (UMF). The region between LMF and UMF forms the FOU [39]. The Karnik–Mendel (KM) iterative algorithm is employed for interval type-reduction, after which the crisp output is obtained by averaging the left and right endpoints. Figure 4 illustrates an example of the membership function of IT2FLS.

Each input variable is categorized into five linguistic levels: Negative Big (NB), Negative small (NS), Zero (Z), Positive Small (PS), and Positive Big (PB). Table 4 shows Linguistic rule base of the IT2FLS.

The fuzzy logic rules are defined as follows:

(39) $$if\;S=L\;\&\;\dot{S}=H\;then\;K=T.$$

(40) $$if\;e=L\;\&\;\dot{e}=H\;then\;\Lambda =T.$$

where *L* and *H* represent labels for input fuzzy subsets, while *T* represents output fuzzy subsets. Specifically, two examples of fuzzy logic rules are given as follows:

(41) $$if\;S=NB\;\&\;\dot{S}=Z\;then\;K=NS$$

(42) $$if\;S=PS\;\&\;\dot{S}=NS\;then\;K=PS$$

In interval type-2 fuzzy systems, defuzzification involves a two-step process consisting of type reduction followed by crisp output computation [40]. Unlike type-1 fuzzy systems, where the output of the inference mechanism is a single fuzzy set, the output of an interval type-2 fuzzy inference system is an interval type-2 fuzzy set. Therefore, a type-reduction step is required to convert this set into an interval type-1 fuzzy set before obtaining a crisp output.

The final crisp output is then obtained by applying a defuzzification operation, typically using the average of the interval endpoints, expressed as:

(43) $$y^{*}=\frac{y_{l}+y_{r}}{2}$$

where $y_{l}$ and $y_{r}$ denote the left and right endpoints of the type-reduced set, respectively. This approach provides a computationally efficient and robust defuzzification mechanism, effectively capturing the uncertainty represented by the footprint of uncertainty while producing a single crisp output suitable for control applications.

The defuzzification range of the fuzzy logic system is established based on the minimum and maximum values of the control parameters obtained from the GWO algorithm, as summarized in Table 4. The output gain *K* is defined using five linguistic terms, namely ZE, PS, PM, PB and PL, which are bounded within the interval of minimum and maximum values determined by GWO in Table 3. Therefore, it yields:

(44) $$K\in \left\{\mathrm{ZE},\mathrm{PS},\mathrm{PM},\mathrm{PB},\mathrm{PL}\right\}\in \left[K_{\min},K_{\max}\right].$$

The corresponding crisp values of *K* are calculated as

(45) $$K=K_{\min}+n\;\Delta K,\;n=0,1,2,3,4,$$

where

(46) $$\Delta K=\frac{K_{\max}-K_{\min}}{4}.$$

Similarly, the defuzzification range for the parameter Λ is defined as

(47) $$\Lambda \in \left\{\mathrm{ZE},\mathrm{PS},\mathrm{PM},\mathrm{PB},\mathrm{PL}\right\}\in \left[\Lambda_{\min},\Lambda_{\max}\right],$$

and the corresponding crisp values are given by

(48) $$\Lambda =\Lambda_{\min}+n\;\Delta \Lambda ,\;n=0,1,2,3,4,$$

with

(49) $$\Delta \Lambda =\frac{\Lambda_{\max}-\Lambda_{\min}}{4}.$$

Here, $K_{\min}$ and $K_{\max}$ denote the minimum and maximum values of the switching gain *K*, while $\Lambda_{\min}$ and $\Lambda_{\max}$ represent the corresponding bounds of the parameter Λ. The optimal values of *K* and Λ obtained by GWO determined in Table 3 are used to define the upper and lower bounds of the IT2FLS membership functions according to Equations (44)–(49). Consequently, the IT2FLS tunes the controller parameters online within ranges determined by the offline optimization process.

Each input variable is represented by five interval type-2 triangular membership functions e.g., NB, NS, Z, PS, and PB, resulting in a total of 25 fuzzy IF–THEN rules. The same rule base is employed for both IT2FLS modules.

The objective of the IT2FLS is to adaptively tune the controller parameters in order to reduce the tracking error while ensuring that the control parameters remain within predefined and physically meaningful boundaries. Accordingly, the fuzzy sets, membership functions, and rule base are designed to provide appropriate decisions for the controller parameters.

### 2.6. Stability Analysis

A Lyapunov candidate function is defined as follows:

(50) $$V=\frac{1}{2}S^{T}S$$

where

(51) V>0∀S≠0,V=0ifS=0

The derivative of the Lyapunov candidate function is given as follows:

(52) $$\dot{V}=S^{T}\dot{S}$$

By substituting Equation (22) into Equation (52), we have

(53) $$\dot{V}=S^{T}M^{-1}\left(q\right)(u-C\left(q,\dot{q}\right)\dot{q}-G\left(q\right)-\tau_{h}-d\left(t\right))-S^{T}{\ddot{q}}_{d}+S^{T}\Lambda \dot{e}$$

From Equation (14), the controller law is u=−KS. Therefore, we have:

(54) $$\dot{V}=S^{T}M^{-1}\left(q\right)(-KS-C\left(q,\dot{q}\right)\dot{q}-G\left(q\right)-\tau_{h}-d\left(t\right))-S^{T}{\ddot{q}}_{d}+S^{T}\Lambda \dot{e}$$

Let Γ be defined as:

(55) $$\Gamma =C\left(q,\dot{q}\right)\dot{q}+G\left(q\right)+\tau_{h}+d\left(t\right)+{\ddot{q}}_{d}-\Lambda \dot{e}$$

then

(56) $$\dot{V}=S^{T}M^{-1}\left(q\right)KS-S^{T}M^{-1}\left(q\right)\Gamma$$

**Remark 1.**  *The lumped uncertainty *Γ *consists of the Coriolis and centrifugal term, gravitational torque, human–robot interaction torque, external disturbances, and bounded reference signals. During rehabilitation, the hip and knee joints operate within predefined ranges of motion, while actuator torque and human interaction forces are limited by the mechanical design and safety constraints of the exoskeleton. Consequently, the dynamic terms $M^{-1}\left(q\right)$, $C(q,\dot{q})$, G(q), $\tau_{h}$ and d(t) remain bounded. Together with Assumptions 1 and 2, there exists a positive constant α such that*

(57) ∥Γ∥≤α

**Remark 2.**  *Since the inertia matrix M(q) is symmetric and positive definite, its inverse is bounded. Therefore, $M^{-1}\left(q\right)$ can be absorbed into the adaptive control gain without loss of generality in the stability analysis. Hence, we have:*

(58) $$∥M^{-1}\left(q\right)\Gamma ∥\leq \rho$$

*where ρ is positive definite constant.*

*By applying the Cauchy–Schwarz inequality, we have*

(59) $$\dot{V}\leq -\lambda_{\min}\left(M^{-1}\left(q\right)K\right){∥s∥}^{2}+\rho ∥s∥.$$

*If the sliding gain satisfies K>ρI, then there exists a constant η>0 such that*

(60) $$\dot{V}\leq -\eta ∥s∥,$$

*Because $\dot{V}$ is negative definite for all s≠0, the reaching condition $s^{T}\dot{s}<0$ is satisfied, ensuring finite-time convergence to s=0.*

*Once the sliding surface is reached, s=0 implies the reduced-order dynamics: $\dot{e}+\Lambda e=0.$ The closed-form solution is $e\left(t\right)=e\left(0\right)e^{-\Lambda t},$ which shows exponential convergence of the tracking error. Thus, the sliding variable reaches zero in finite time, and the tracking error converges exponentially to zero, proving the global stability of the control system.*

**Remark 3.**  *The stability proof is established under the assumptions that the system uncertainties, human interaction torques, and external disturbances remain bounded during rehabilitation. These assumptions are reasonable for RLLE because the joints operate within prescribed ranges of motion and are subject to safety constraints on actuator torque and patient interaction forces. However, the theoretical stability guarantee may not strictly hold under extreme operating conditions, such as severe external impacts, actuator saturation, or movements outside the designed rehabilitation range. In such cases, tracking performance may degrade, although the proposed adaptive controller is expected to retain practical robustness.*

## 3. Experimental Verification

**Figure 5:**
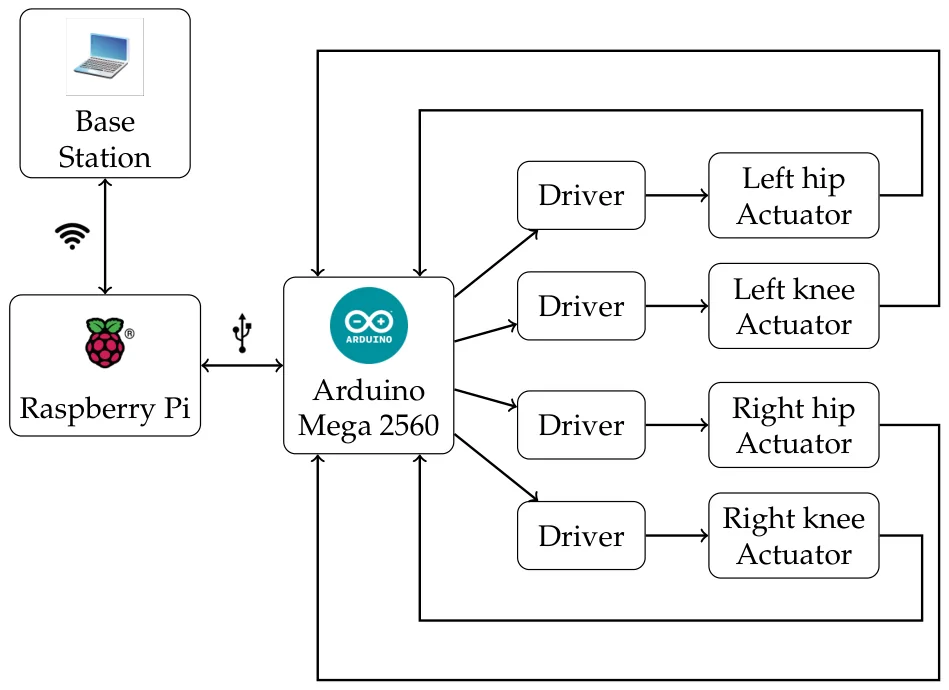
Hardware structure of lower-limb exoskeleton actuation system.

Each joint of the exoskeleton is a revolute joint that is driven by a 12 V brushed DC motor and a worm gearbox. The generated torque is transmitted to a worm gearbox by gear ratio of 32. A quadrature encoder is located at the DC motor rotary shaft to record the angle of each joint by a resolution of 6400 pulses per rotation. An Arduino Mega 2560 is utilized to control the DC motor and capture the data from the encoder and regulate the input voltage and direction of actuators based of the determined data from the control system. Figure 5 illustrates the structure of hardware used for the control system of the lower-limb exoskeleton.

A DC motor driver is used to convert the direction and voltage received from Arduino to the actuators of each joint. The actual trajectories are captured by encoders as the feedback of the control system. A Raspberry Pi 5 8 GB is employed to communicate with Arduino and the base station. A Robot Operating System (ROS) is used as the master and client for this communication. The control system is programmed in Python 3.6 language and ran in the base station, which is a PC with Intel core i7 CPU and 32 GB RAM. The control loop was executed at 100 Hz, which is sufficient for lower-limb rehabilitation control applications.

In the experimental evaluation, each joint moved in the Range of Motion (RoM), in which each joint moved individually to evaluate the performance of the control system on each joint separately. In this paper, four healthy subjects are required to wear the RLLE. The features of the subjects are given in Table 5.

**Figure 6:**
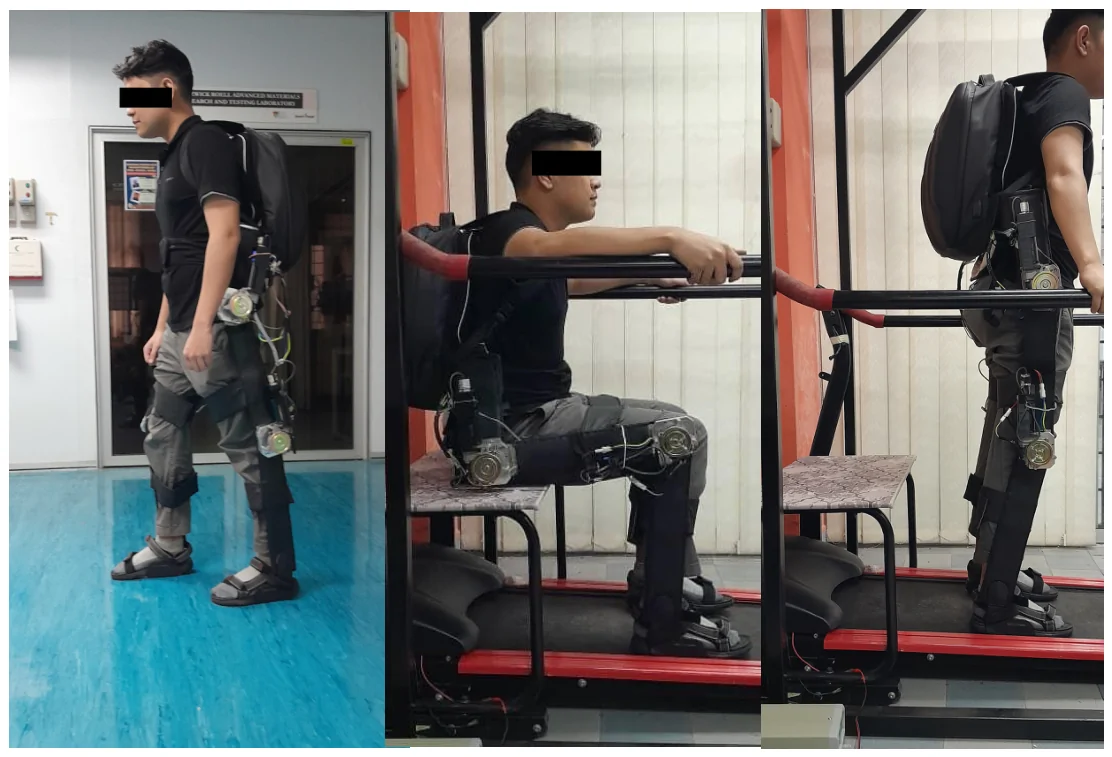
RLLE worn by a healthy human subject.

Figure 6 illustrates the RLLE worn by a healthy human subject in the sitting and standing positions.

### Results and Analysis

**Figure 7:**
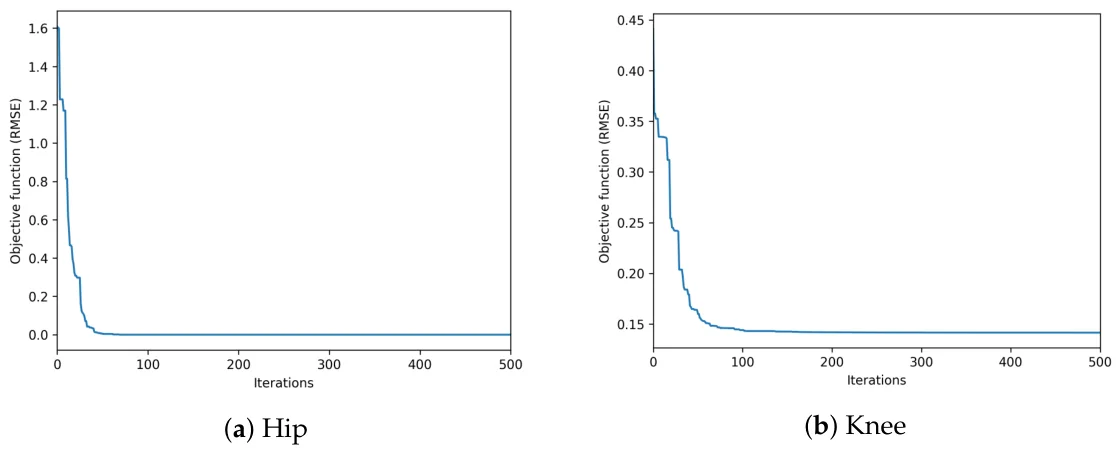
Convergence histories of the GWO objective function for the hip and knee joints. The curves represent the best objective value obtained at each iteration.

In order to evaluate the performance of the tuning capability of GWO, the transfer function of hip and knee is applied in a closed-loop control system to tune its parameters. Figure 7 shows the convergence graphs of the objective functions evaluated for the best wolves in each iteration.

In order to evaluate the convergence characteristics of the GWO, the transfer functions of the hip and knee joints were optimized using the objective functions defined in Equations (12) and (13). Figure 7 presents the convergence histories of the objective function corresponding to the best wolf at each iteration. As observed, the objective function decreases rapidly during the early iterations and approaches a stable minimum after approximately 50 iterations for the hip joint and 80 iterations for the knee joint. Beyond these points, only marginal improvements are observed, indicating that the selected maximum of 500 iterations is sufficient to ensure convergence while avoiding unnecessary computational effort. Since the optimization problem involves only two controller parameters (*K* and Λ), a population size of five wolves provides an appropriate balance between search diversity, computational efficiency, and optimization quality. In order to evaluate the sensitivity of the GWO to the population size when determining the controller parameters, the number of wolves was varied while keeping the maximum number of iterations fixed at 500. Table 6 presents the RMS tracking errors obtained for the hip and knee joints under different population sizes.

As shown in Table 6, increasing the population size beyond five wolves resulted in only marginal improvements in the RMS tracking errors for both the hip and knee joints. Therefore, a population size of five was selected, as it provides an appropriate balance between optimization performance and computational efficiency.

**Figure 8:**
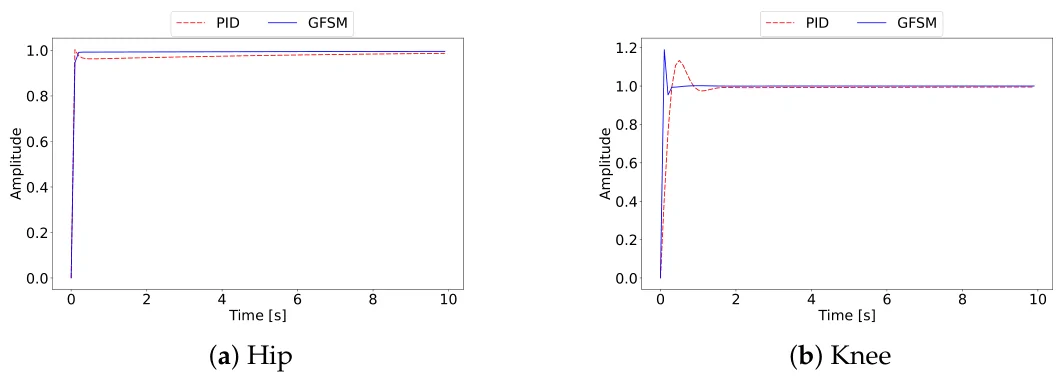
Comparison of step response for GFSM and PID controller.

The transfer functions presented in Table 2 and the average controller parameters obtained using GWO, as listed in Table 3, were incorporated into the closed-loop control system of the hip and knee joints. Figure 8 compares the step responses of the proposed GFSM controller and the conventional PID controller.

For the hip joint, the GFSM controller achieved a settling time of 0.2 s, whereas the PID controller required 1.8 s to reach steady-state. Similarly, for the knee joint, the settling times were 0.3 s and 1.2 s for the GFSM and PID controllers, respectively. These step-response results clearly demonstrate the superior performance of the GFSM controller over the PID controller, particularly in terms of settling time.

For the evaluation of the GFSM controller in a prototype exoskeleton, the following desired trajectories are defined based on the limitation of human lower-limb joints movement as follows [41]:

(61) $$q_{d}^{RH}\left(t\right)=max\left(15sin\left(ftt+\pi \right),30sin\left(ft+\pi \right)\right)$$

(62) $$q_{d}^{RK}\left(t\right)=min\left(0,15sin\left(ft+\pi \right)\right)$$

(63) $$q_{d}^{LH}\left(t\right)=max\left(15sin\left(ft\right),30sin\left(ft\right)\right)$$

(64) $$q_{d}^{LK}\left(t\right)=min\left(0,15sin\left(ft\right)\right)$$

where $q_{d}^{RH}$, $q_{d}^{RK}$, $q_{d}^{LH}$, and $q_{d}^{LK}$ represent the desired trajectories for the right hip, right knee, left hip, and left knee, respectively. The variables *t* and *f* denote the time that has passed and the frequency.

Equations (61) and (63) are defined for right and left hip angular movement to 30 degrees during flexion and 15 degrees during extension. For the right and left knee extension, we set 15 degrees for knee flexion and 0 degrees for extension as mentioned in Equations (62) and (64).

**Figure 9:**
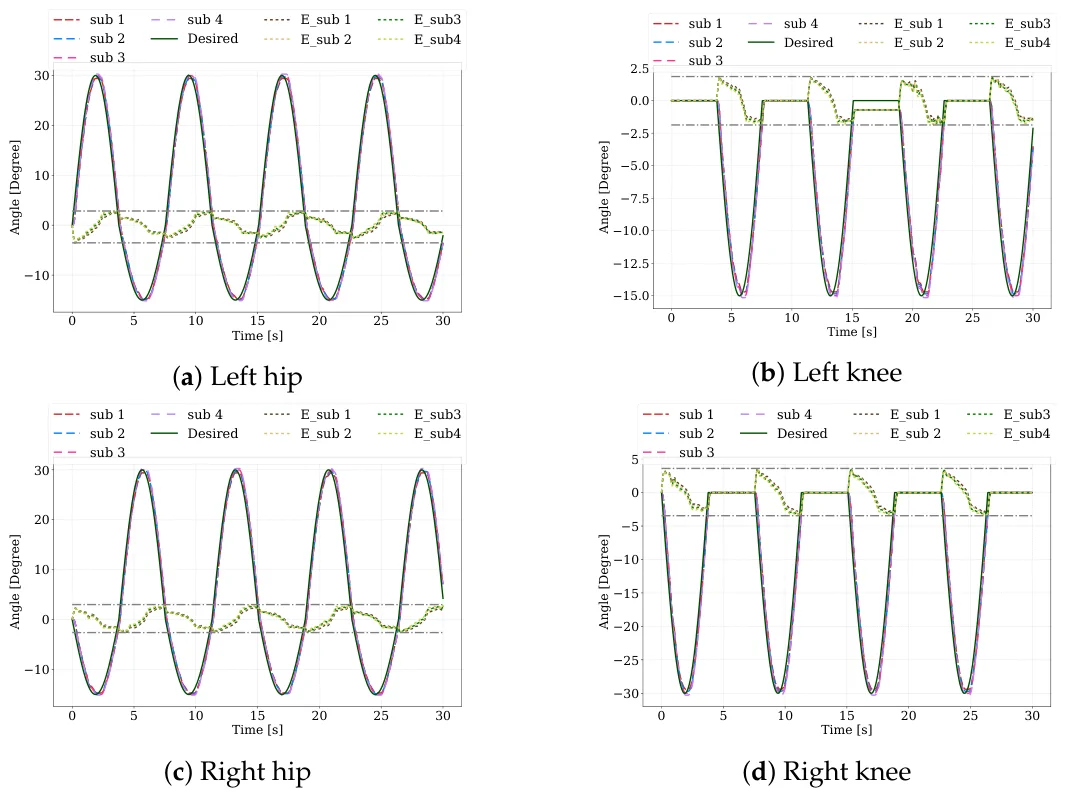
Trajectory tracking and error for hip and knee.

Figure 9 illustrates the trajectory tracking performance of GFSM for hip and knee.

**Figure 10:**
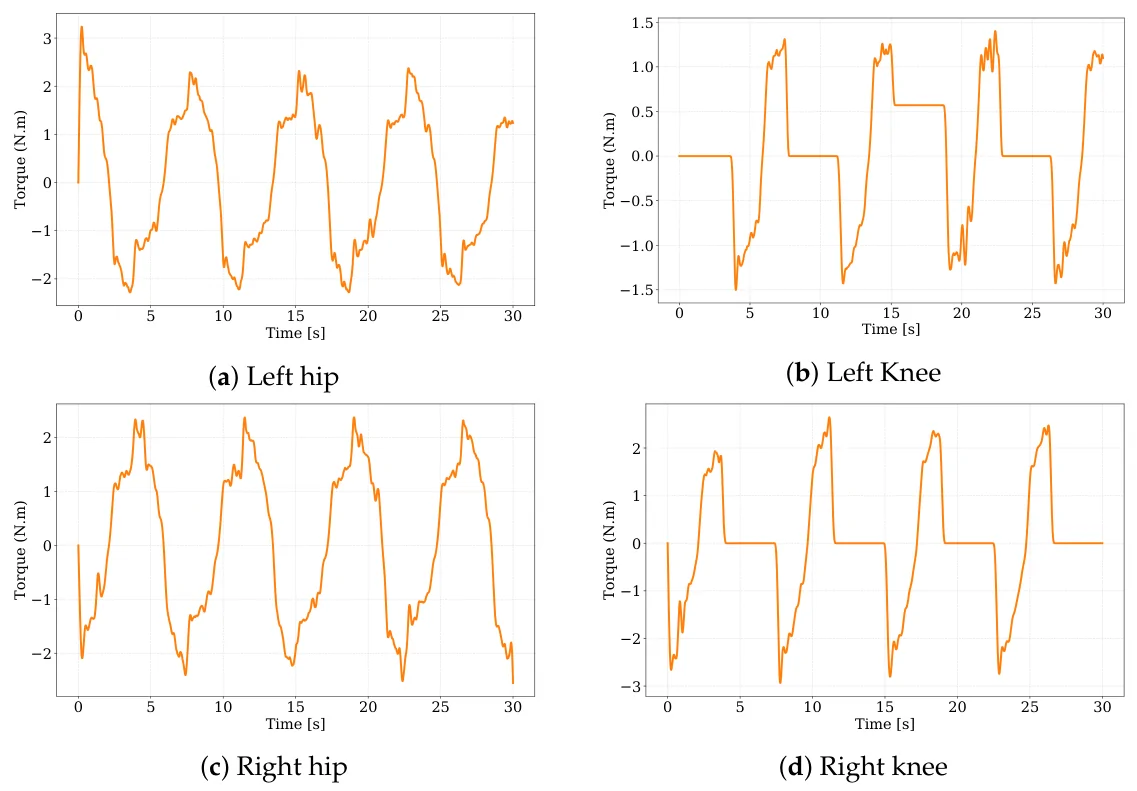
GFSM controller output for hip and knee.

The analysis of Figure 9 indicates that the tracking error *e* remains within a bounded range, representing the effective performance of the adaptive GFSM controller. Furthermore, Figure 10 shows the output of the controller for each joint of the first healthy human subject.

**Figure 11:**
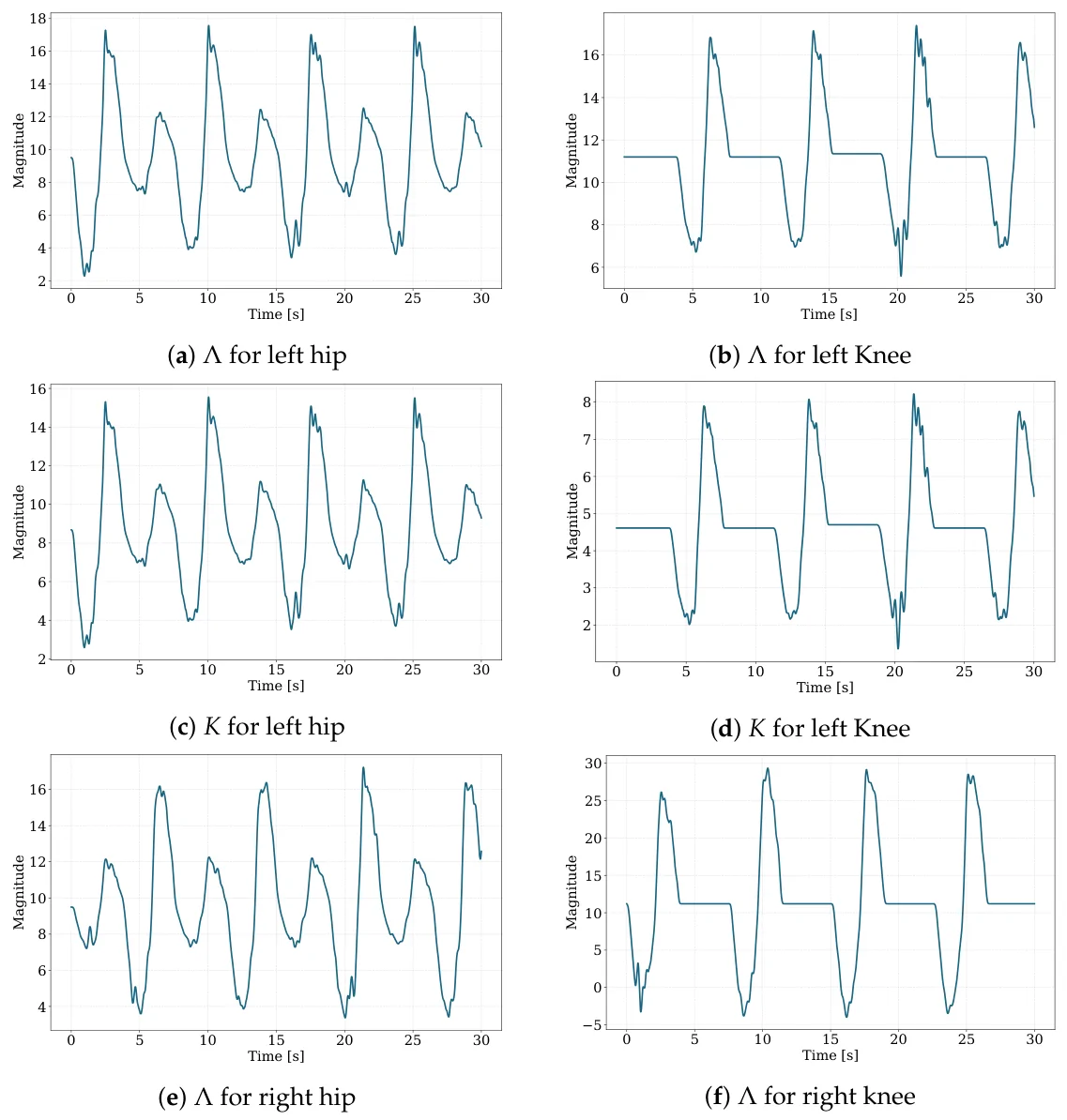

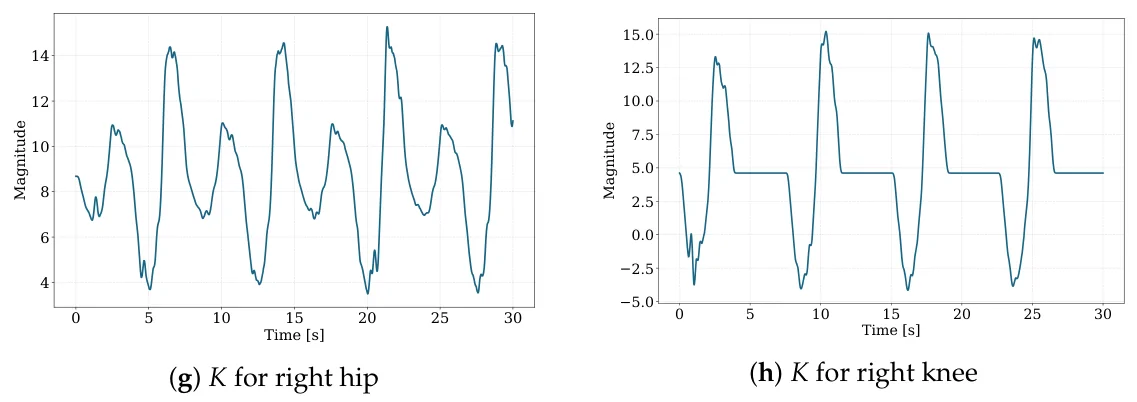
Parameters of GFSM controller for hip and knee.

In Figure 10, the controller output stays within a stable and limited range. As the desired trajectory shifts, the controller output varies. This showcases the GFSM control system’s effective adaptability. Furthermore, Figure 11 illustrates the changes in the controller parameters for each joint tested for the first healthy human subject.

Table 3 illustrates that the controller parameters fluctuate between the minimum and maximum values of *K* and Λ, as determined by the fuzzy logic systems. These systems adjusted the parameters within the range established by GWO for the purpose of defuzzification.

The RMS of the tracking error of the first healthy human subject was determined and is shown in Table 7. This table includes the GFSM controller, PID controller from [42], Lyapunov Adaptive and Swarm-Fuzzy Logic Controller (LASFC) from [43], Adaptive Fuzzy Controller (AFC) from [44] and fuzzy controller from [45].

The proposed GFSM controller achieves the lowest or comparable RMS values across all joints.

For the left hip joint, GFSM obtains 0.022, which is equal to the Fuzzy adaptive controller and improves the performance by approximately 68.6% compared to PID, 24.1% compared to LASFC, and 81.8% compared to AFC. For the left knee joint, GFSM achieves 0.017, which is the lowest value among all controllers. This corresponds to improvements of about 73.4% over PID, 51.4% over LASFC, 34.6% over the Fuzzy adaptive method, and 82.9% over AFC. For the right hip joint, GFSM records 0.021, showing improvements of approximately 70.8% compared to PID, 30.0% compared to LASFC, 12.5% compared to the Fuzzy adaptive controller, and 84.1% compared to AFC. Similarly, for the right knee joint, GFSM achieves 0.018, which represents improvements of about 77.8% over PID, 51.4% over LASFC, 5.3% over the Fuzzy adaptive method, and 80.6% over AFC.

Overall, GFSM consistently provides significantly lower steady-state RMS errors compared to PID, LASFC, and AFC, and achieves equal or improved performance relative to the Fuzzy adaptive controller. These results demonstrate the superior steady-state accuracy and enhanced control effectiveness of the proposed GFSM approach across all lower-limb joints.

The obtained results demonstrate that the proposed GFSM controller provides accurate trajectory tracking while maintaining robustness against model uncertainties and external disturbances, making it suitable for lower-limb rehabilitation applications. The integration of the IT2FLS with the adaptive sliding mode controller enables the control system to effectively compensate for parameter variations and unmodeled dynamics arising from human–robot interaction, thereby reducing tracking errors and improving control stability. From a computational perspective, the GWO is employed only during the offline tuning stage to determine the controller parameters, while the online implementation requires only the execution of the optimized controller, resulting in a computationally efficient real-time control strategy. This separation between offline optimization and online control makes the proposed approach suitable for embedded rehabilitation platforms with limited computational resources. Although the experimental results obtained from the developed RLLE validate the effectiveness of the proposed controller, several limitations remain. The experiments were conducted under controlled laboratory conditions and therefore may not fully represent the dynamic characteristics of patients with different levels of motor impairment. Furthermore, the stability analysis assumes bounded disturbances and human interaction forces, which may not hold under extreme operating conditions. Future work will focus on clinical validation involving patients with gait impairments, adaptive personalization of controller parameters according to patient recovery stages, and evaluation of the controller under more complex rehabilitation scenarios involving varying walking speeds and uneven terrains.

## 4. Conclusions

This paper presents a GFSM controller, which is a combination of GWO with an IT2FLS to tune the parameters of a sliding-mode controller in offline and real-time loops for an RLLE. The GFSM controller was established to reduce the steady-state error of the joint trajectory tracking within a bounded range. The range of the IT2FLS defuzzification was determined by optimal tuning of the controller parameters for the RLLE. The Lyapunov function was derived to prove the stability of the control system.

The performance of the proposed GFSM controller was evaluated through experiments on a four-DoF rehabilitation lower-limb exoskeleton worn by a healthy human subject. The experimental results were compared with several advanced control methods, including PID, LASFC, fuzzy adaptive control, and AFC. The proposed GFSM controller demonstrated superior tracking performance, achieving average RMS error reductions of 68.6% compared with PID, 24.1% compared with LASFC, and 81.8% compared with AFC. These results confirm the effectiveness and robustness of the proposed GFSM approach for rehabilitation lower-limb exoskeleton control. The proposed GESM controller further contributes to biomimetic rehabilitation robotics by enabling the RLLE to achieve robust and adaptive tracking of natural lower-limb gait trajectories.

Despite the promising results, this study has several limitations. The experimental validation was conducted using a healthy human subject, and therefore the performance of the proposed controller has not yet been evaluated on patients with neurological impairments undergoing rehabilitation. In addition, the experiments focused on trajectory tracking under controlled rehabilitation conditions, while more complex activities involving varying walking speeds, uneven terrains, and different rehabilitation scenarios were not investigated. Future work will focus on clinical validation involving a larger number of participants, including post-stroke patients, as well as extending the proposed control framework to adaptive gait assistance under diverse rehabilitation conditions. Additionally, algorithms and methods will be investigated to adjust the optimal parameters of the control system by integrating a disturbance observer.

## Figures and Tables

**Table 1:** Properties of the RLLE.

Joints	DoF	Motion	Property	Link Length	Rotation Range (Degree)
Left/ Right hip	2	flexion & extension	Active	40 cm	[−45, 30]
Left/ Right knee	2	flexion & extension	Active	41 cm	[−40, 0]

**Table 2:** Parameters of the mathematical model for hip and knee.

	$b_{i}$	$a_{3_{i}}$	$a_{2_{i}}$	$a_{1_{i}}$	$a_{0_{i}}$
Hip (i=1)	26.459	0.002	0.237	1.607	4.661
Knee (i=2)	25.991	0.002	0.065	0.566	1.733

**Table 3:** Numerical comparison of GWO, GA, PSO, and WOA over 10 independent runs for hip and knee joints.

Method	Hip Joint			Knee Joint		
	K	Λ	RMS (rad)	K	Λ	RMS (rad)
GA	14.22	12.63	0.091	3.91	2.68	0.197
	5.81	6.56	0.125	7.01	6.72	0.123
	8.47	9.67	0.103	2.81	7.15	0.122
	15.79	17.21	0.077	2.17	10.16	0.103
	16.01	17.73	0.076	2.80	7.74	0.117
	4.59	4.46	0.152	2.17	8.50	0.113
	11.20	10.11	0.100	2.18	9.90	0.105
	3.22	4.11	0.160	2.79	12.53	0.091
	8.59	7.23	0.119	3.79	16.04	0.081
	3.58	5.59	0.136	4.47	17.95	0.077
Average	9.148	9.530	0.114	3.410	9.937	0.113
Standard Deviation	5.106	4.831	0.030	1.538	4.565	0.034
Maximum	16.01	17.73	0.160	7.01	17.95	0.197
Minimum	3.22	4.11	0.076	2.17	2.68	0.077
GWO	6.46	15.98	0.082	2.00	5.92	0.131
	5.43	3.53	0.170	2.26	8.25	0.119
	3.27	17.83	0.078	6.60	9.01	0.106
	15.10	10.71	0.097	5.97	9.12	0.105
	3.09	6.53	0.127	9.01	13.56	0.086
	9.96	13.38	0.087	6.19	17.07	0.077
	11.08	7.52	0.116	4.52	9.51	0.103
	4.51	16.05	0.081	5.62	7.60	0.115
	6.02	12.51	0.091	6.56	13.76	0.086
	5.58	14.73	0.084	3.57	7.5	0.116
Average	7.050	11.877	0.101	5.230	10.130	0.104
Standard Deviation	3.832	4.703	0.029	2.162	3.501	0.017
Maximum	15.10	17.83	0.170	9.01	17.07	0.131
Minimum	3.09	3.53	0.078	2.00	5.92	0.077
PSO	2.24	5.09	0.0145	2.52	2.5	0.2
	2.01	4.48	0.155	3.92	9.55	0.103
	1.96	6.39	0.135	3.83	6.46	0.125
	2.67	5.54	0.138	3.83	6.15	0.128
	2.72	7.90	0.117	2.37	7.25	0.119
	3.51	4.91	0.146	1.52	3.76	0.166
	5.75	7.22	0.119	3.21	5.18	0.140
	4.13	5.68	0.135	3.26	7.35	0.118
	2.25	4.46	0.155	1.85	3.51	0.176
	1.44	3.03	0.194	4.16	6.35	0.126
Average	2.868	5.470	0.131	3.047	5.806	0.140
Standard Deviation	1.280	1.425	0.046	0.931	2.111	0.031
Maximum	5.75	7.90	0.194	4.16	9.55	0.200
Minimum	1.44	3.03	0.0145	1.52	2.50	0.103
WOA	5.92	0.43	0.497	0.31	6.98	0.141
	5.08	0.57	0.446	1.63	0.56	0.455
	7.04	0.81	0.361	3.95	1.02	0.332
	8.11	0.69	0.389	10.54	8.17	0.118
	5.94	0.31	0.579	6.29	1.20	0.305
	2.73	1.08	0.319	6.47	3.58	0.171
	3.58	0.61	0.424	7.63	3.46	0.176
	4.10	0.65	0.419	3.28	2.55	0.203
	8.84	0.18	0.719	5.36	7.05	0.119
	7.29	0.64	0.397	7.51	2.61	0.195
Average	5.863	0.597	0.455	4.697	3.718	0.222
Standard Deviation	2.003	0.253	0.117	3.062	2.745	0.109
Maximum	8.84	1.08	0.719	10.54	8.17	0.455
Minimum	2.73	0.18	0.319	0.31	0.56	0.118

**Table 4:** Linguistic rule base of the IT2FLS.

	$\dot{S}$ & $\dot{e}$	NB	NS	Z	PS	PB
*S* & *e*						
NB		NB	NB	NS	Z	Z
NS		NB	NS	Z	PS	PS
Z		NS	Z	Z	Z	PS
PS		Z	PS	Z	PS	PB
PB		Z	PS	PS	PB	PB

**Table 5:** Features of four healthy human subjects who wore RLLEs.

	Height (cm)	Weight (kg)	Age	Gender
subject 1	171	69	23	Male
subject 2	165	68	47	Female
subject 3	170	75	35	Male
subject 4	178	96	37	Male

**Table 6:** Sensitivity analysis of the GWO population size with a fixed maximum iteration of 500.

Population	Hip RMS (rad)	Knee RMS (rad)
5	0.101	0.104
10	0.100	0.103
20	0.100	0.103

**Table 7:** RMS error for steady-state error.

Controllers	Left Hip	Left Knee	Right Hip	Right Knee
GFSM	0.022	0.017	0.021	0.018
PID in [42]	0.070	0.064	0.072	0.081
LASFC in [43]	0.029	0.035	0.030	0.037
Fuzzy adaptive in [45]	0.022	0.026	0.024	0.019
AFC in [44]	0.1209	0.0992	0.1319	0.0929

## Data Availability

The original contributions presented in this study are included in the article. Further inquiries can be directed to the corresponding authors.
